# Protein profile analysis of Jilin white goose testicles at different stages of the laying cycle by DIA strategy

**DOI:** 10.1186/s12864-024-10166-9

**Published:** 2024-04-01

**Authors:** Yupu Song, Jingyun Ma, Qiuyuan Liu, Ichraf Mabrouk, Yuxuan Zhou, Jin Yu, Fengshuo Liu, Jingbo Wang, Zhiye Yu, Jingtao Hu, Yongfeng Sun

**Affiliations:** 1 College of Animal Science and Technology, Jilin Agricultural University, 130118 Changchun, China; 2 Key Laboratory for Animal Production, Product Quality and Safety of Ministry of Education, 130118 Changchun, China

**Keywords:** Jilin white goose, Testis, Proteomics, Poultry, Sperm

## Abstract

**Background:**

Jilin white goose is an excellent local breed in China, with a high annual egg production and laying eggs mainly from February to July each year. The testis, as the only organ that can produce sperm, can affect the sexual maturity and fecundity of male animals. Its growth and development are affected and regulated by a variety of factors. Proteomics is generally applied to identify and quantify proteins in cells and tissues in order to understand the physiological or pathological changes that occur in tissues or cells under specific conditions. Currently, the female poultry reproductive system has been extensively studied, while few related studies focusing on the regulatory mechanism of the reproductive system of male poultry have been conducted.

**Results:**

A total of 1753 differentially expressed proteins (DEPs) were generated in which there were 594, 391 and 768 different proteins showing differential expression in three stages, Initial of Laying Cycle (ILC), Peak of Laying Cycle (PLC) and End of Laying Cycle (ELC). Furthermore, bioinformatics was used to analyze the DEPs. Gene ontology (GO) enrichment, Clusters of Orthologous Groups (COG), Kyoto Encyclopedia of Genes and Genomes (KEGG) and protein-protein interaction (PPI) network analysis were adopted. All DEPs were found to be implicated in multiple biological processes and pathways associated with testicular development, such as renin secretion, Lysosomes, SNARE interactions in vesicle trafficking, the p53 signaling pathway and pathways related to metabolism. Additionally, the reliability of transcriptome results was verified by real-time quantitative PCR by selecting the transcript abundance of 6 selected DEPs at the three stages of the laying cycle.

**Conclusions:**

The funding in this study will provide critical insight into the complex molecular mechanisms and breeding practices underlying the developmental characteristics of testicles in Jilin white goose.

**Supplementary Information:**

The online version contains supplementary material available at 10.1186/s12864-024-10166-9.

## Background

Goose is widely raised in China and its domestication can be traced back to around 7000 BC in the middle and lower reaches of the Yangtze River, making it the oldest domesticated poultry species in history [1]. The Jilin white goose is a high-quality local goose species in China, domesticated from *Anser cygnoides*, which not only tolerant to coarse grains and cold but also has a high egg-laying performance [2]. Simultaneously, we discovered that the egg-laying of Jilin white goose exhibits seasonal characteristics [3], which mainly occur between February and July each year.

It has been widely reported that the reproductive ability of male poultry has an important impact on overall poultry production [3]. As the only organ capable of producing sperm, the testis plays an important role in regulating the sexual maturity and fertility of male poultry [4]. Furthermore, testicles are mainly composed of spermatogenic cells, Leydig cells and supporting cells at all levels, and their growth and development are affected and regulated by a variety of factors [5, 7]. The emergence of proteomics provides solutions to identify and quantify protein expression levels, amino acid sequences, and protein-protein interactions in cells or tissues [8, 9]. It can also reveal the physiological or pathological changes of cells or tissues under certain conditions, thereby elucidating the complex regulatory mechanisms of organisms.

At present, there are many studies on the reproductive system of female poultry, but few related studies on the regulatory mechanism of the reproductive system of male poultry were published [10, 12]. Therefore, studying the differences of protein expression during testicular growth and development of Jilin white goose at different stages of the laying cycle will help clarify the cyclic changes in the testes during the reproductive cycle in poultry, and further improve the production performance of Jilin white goose and accelerate its breeding process.

## Results

### HE staining observations of testicular tissue at different stages of laying cycle

**Fig. 1:**
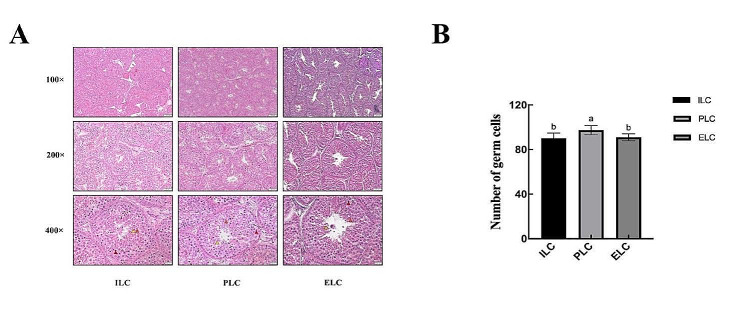
Histological observations and the counting the number of germ cells located in single seminiferous tubules of the Jilin white goose testicular tissue morphology at different stages of laying cycle. **(A)** HE staining of the testis (The first line: Observations of the Jilin white goose testicular tissue morphology at different stages of laying cycle under 100 times magnification, bar = 100 μm; The second line: Observations of the Jilin white goose testicular tissue morphology at different stages of laying cycle under 200 times magnification, bar = 50 μm; The third line: Observations of the Jilin white goose testicular tissue morphology at different stages of laying cycle under 400 times magnification, bar = 20 μm);red triangle refers to primary spermatocytes; orange triangle refers to sperm cells refers to yellow triangle: sperm). **(B)** the measurement of the number of germ cells located in single seminiferous tubules. Different letters represent significant different

HE staining was used to observe the morphology of testicular tissue at three laying cycle stages: ILC (Initial of Laying Cycle), PLC (Peak of Laying Cycle) and ELC (End of Laying Cycle). The results are shown in Fig. 1. The testicles at the three stages have normal morphology, and the germ cells are arranged regularly, with spermatogonia, sperm cells, and sperm are distinguishable in all the observations. Furthermore, we noticed that the number of germ cells was significantly higher in the PLC stage and in the range of 80 cells (Fig. 1B).

### Quantitative protein detection

Traditional mass spectrometry and data-dependent acquisition (DDA) were used to create and analyze ILC, PLC, and ELC spectral libraries of Jilin white goose testicular tissue. As a result, a total of 1753 of DEPs were identified. We then used the data-independent acquisition (DIA) method to collect mass spectrometry data. Proteins with log_2_fold-change ≥ 0.58 or ≤ -0.58 and *P* < 0.05 were identified as significantly differentially expressed proteins (DEP). Three sets of DEPs were obtained, 594 (PLC vs. ILC, 303 up-regulated, 291 down-regulated), 391 (ELC vs. ILC, 84 up-regulated, 307 down-regulated) and 768 (ELC vs. PLC, 319 up-regulated, 449 down-regulated). Table 1 briefly summarizes the number of proteins for each sample.

**Table 1:** Statistics of DEPs identified by different groups

Groups	Total Trusted Proteins	Total DEPs	Up regulated	Down regulated
PLC: ILC	5899	594	303	291
ELC: ILC	5419	391	84	307
ELC: PLC	5601	768	319	449

**Fig. 2:**
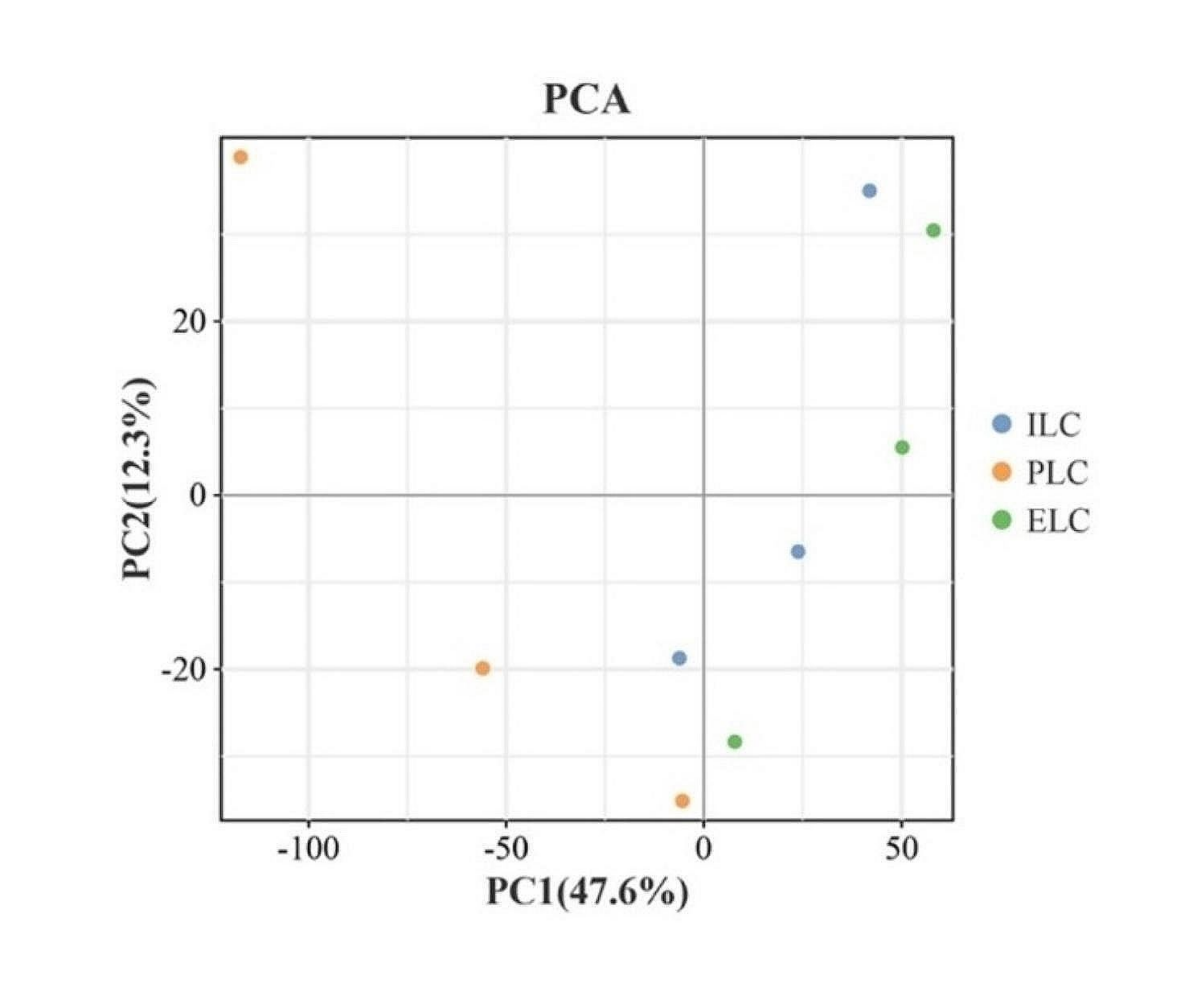
Principal Component Analysis (PCA) plot of proteins identified in the testicular proteome of Jilin White Geese at three stages of the laying cycle. The colored points in the graph indicate the individual samples

We further analyzed the relationship between the samples using the Principal Component Analysis (PCA) and choose the top two principal components to plot the results (Fig. 2). The PCA results demonstrated that for PC1, the ILC, PLC and ELC groups were relatively close to each other. For the PC2, the samples were relatively separate from each other and in the remaining three groups can still be obtained with good separation. As noticed the three sets of samples were separated and the PCL group samples were significantly distant from the other two groups of samples indicating that the subsequent differences obtained between groups could be a relatively realistic reflection of the biological differences between groups.

### GO enrichment analysis of the differentially expressed proteins

**Fig. 3:**
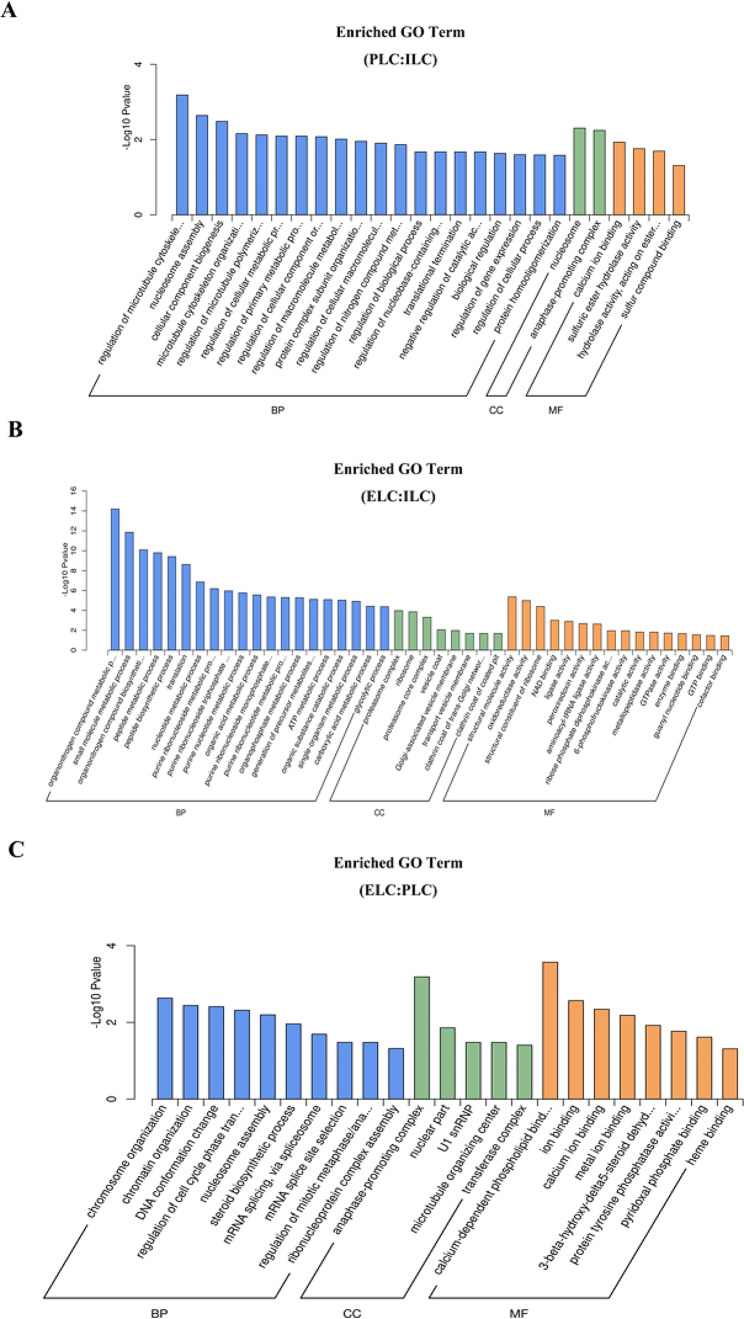
GO enrichment analysis of differentially expressed proteins (DEPs) in Jilin white goose testicles at different stages of the laying cycle. (**A**-**C**) shows the comparison group of ILC, PLC and ELC. The results are elaborated into three major groups: Biological process, cellular component and molecular function. The Y-axis shows the percentage of proteins, while the X-axis displays the second level term of the gene ontology

To assess the functional significance of all identified proteins, gene ontology (GO) annotation analysis was performed using Blast2GO software. The results are shown in Fig. 3. The DEPs were classified into three main GO categories based on the GO annotation: Biological process (‘regulation of microtubule cytoskeleton organization’, ‘organonitrogen compound metabolic process’ and ‘chromosome organization’), cellular component (‘nucleosome’, ‘proteasome complex’ and ‘anaphase-promoting complex’) and molecular function (‘calcium ion binding’, ‘structural molecule activity’ and ‘calcium-dependent phospholipid binding’).

### Function classification in clusters of Orthologous Groups of Proteins (COG)

**Fig. 4:**
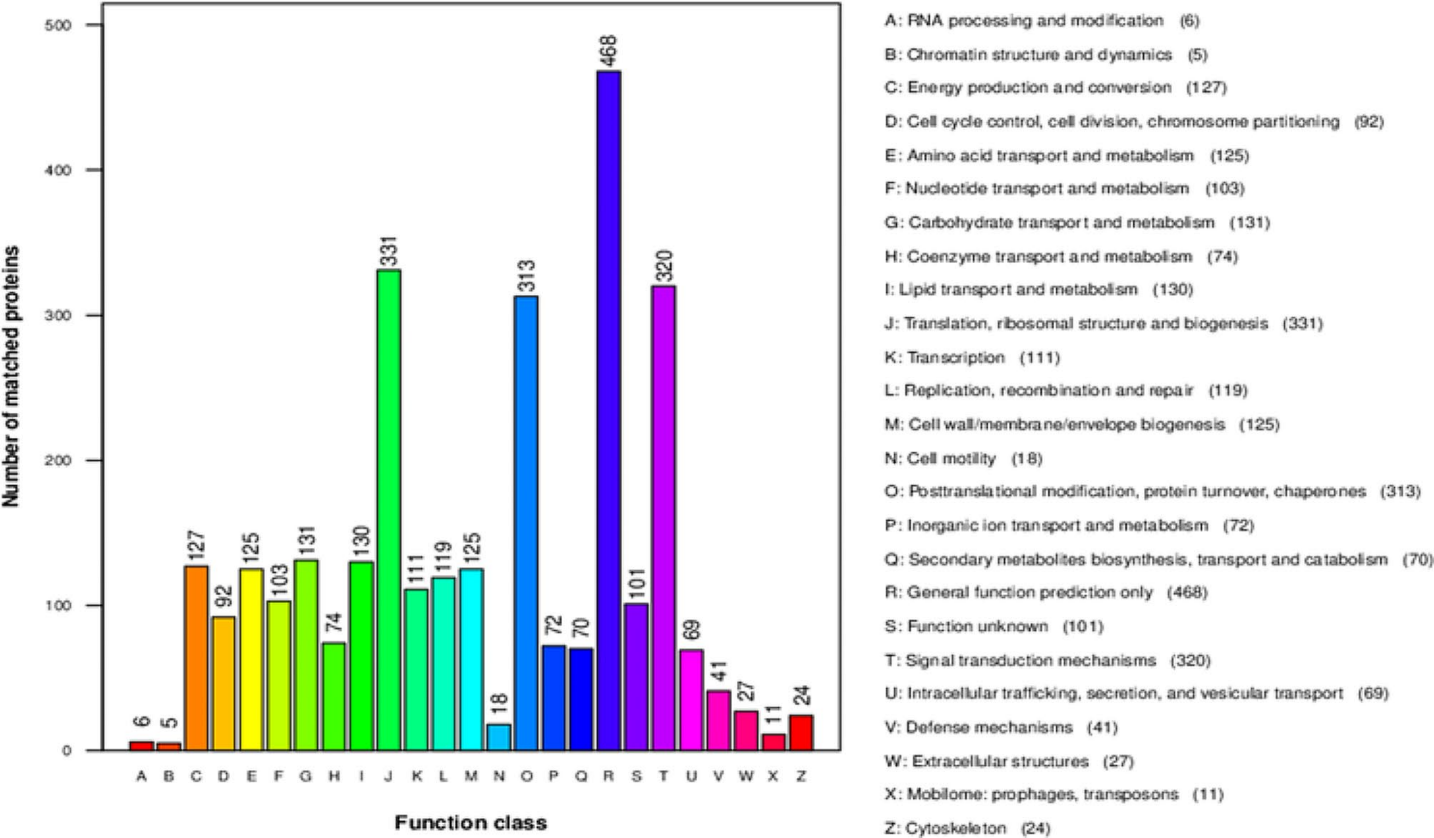
Function classification in Clusters of Orthologous Groups of Proteins (COG**).** (All proteins were aligned to the COG database to predict possible functions. A total of 3013 putative proteins were functionally classified into 25 groups. Capital letters on the X-axis shows the COG categories as listed on the right of the histogram and the Y- axis shows the number of matched proteins)

Next, we predicted the potential functions of the identified DEPs using the Clusters of Orthologous Groups of proteins (COG). The functional classification of protein orthologous groups is shown in Fig. 4. The largest category was ‘General function prediction only’ (468 COG annotations, 15,53% of 3013), following by ‘Translation, ribosomal structure and biogenesis’ (331, 10,99%), ‘Signal transduction mechanisms’ (320, 10,62%) and ‘Posttranslational modification, protein turnover, chaperones’ (313, 10,39%). In addition, only 101 (3,35%) COG annotations belonged to the ‘Function unknown’ category.

### KEGG pathway enrichment analysis of DEPs

**Fig. 5:**
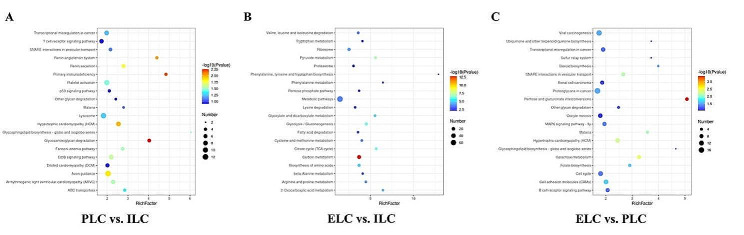
KEGG pathway enrichment analysis of differentially expressed proteins (DEPs) in Jilin white goose testicles at different stages of the laying cycle. (**A**-**C** shows the comparison group of ILC, PLC and ELC. The abscissa represents the number of enriched proteins, and the ordinates represent signal pathways)

The Kyoto Encyclopedia of Genes and Genomes (KEGG) annotation aids us to better comprehend the changes in signaling pathways. The results are shown in Fig. 5. The most strongly represented pathways were ‘Renin secretion’, ‘Lysosomes’, followed by ‘SNARE interactions in vesicular transport’ and ‘p53 signaling pathway’ using ILC as reference. For the ELC vs. ILC comparison group, the various DEPs detected in our testis samples were enriched in ‘Metabolic pathway’, ‘Carbon metabolism’, ‘Biosynthesis of amino acids’ and ‘Citrate cycle (TCA cycle)’. On the other hand, using PLC as a reference, DEPs were mainly annotated in the ‘MAPK signaling pathway’, ‘Ras signaling’, ‘cell cycle’, ‘cell adhesion molecules (CAMs)’ and ‘SNARE interactions in vesicular transport’.

### Protein-protein interaction (PPI) network analysis

**Fig. 6:**
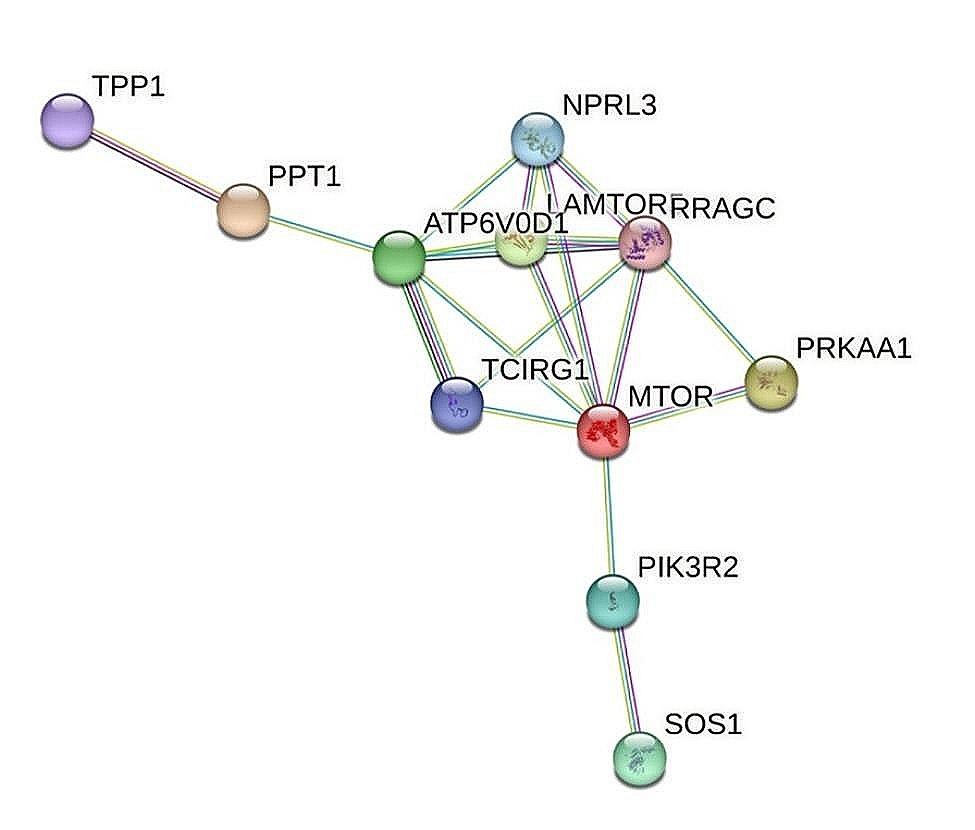
The protein-protein interaction (PPI) network analysis of differentially expressed proteins. (Proteins are represented with color nodes and interactions are represented with edges)

In order to reveal the protein interaction network during the testicular development of Jilin white goose, DEPs were used to construct a protein interaction network. As shown in Fig. 6, the protein-protein interactions are multiform and eleven interacting proteins related to the key pathways were detected: TPP1, PPT1, NPRL3, ATP6V0D1, LAMTOR5, RRAGC, TCIRG1, MTOR, PRKAA1, PIK3R2 and SOS1, which are involved in different pathways related to testicular development.

### Validation of selected DEPs expression results by qRT-PC

**Fig. 7:**
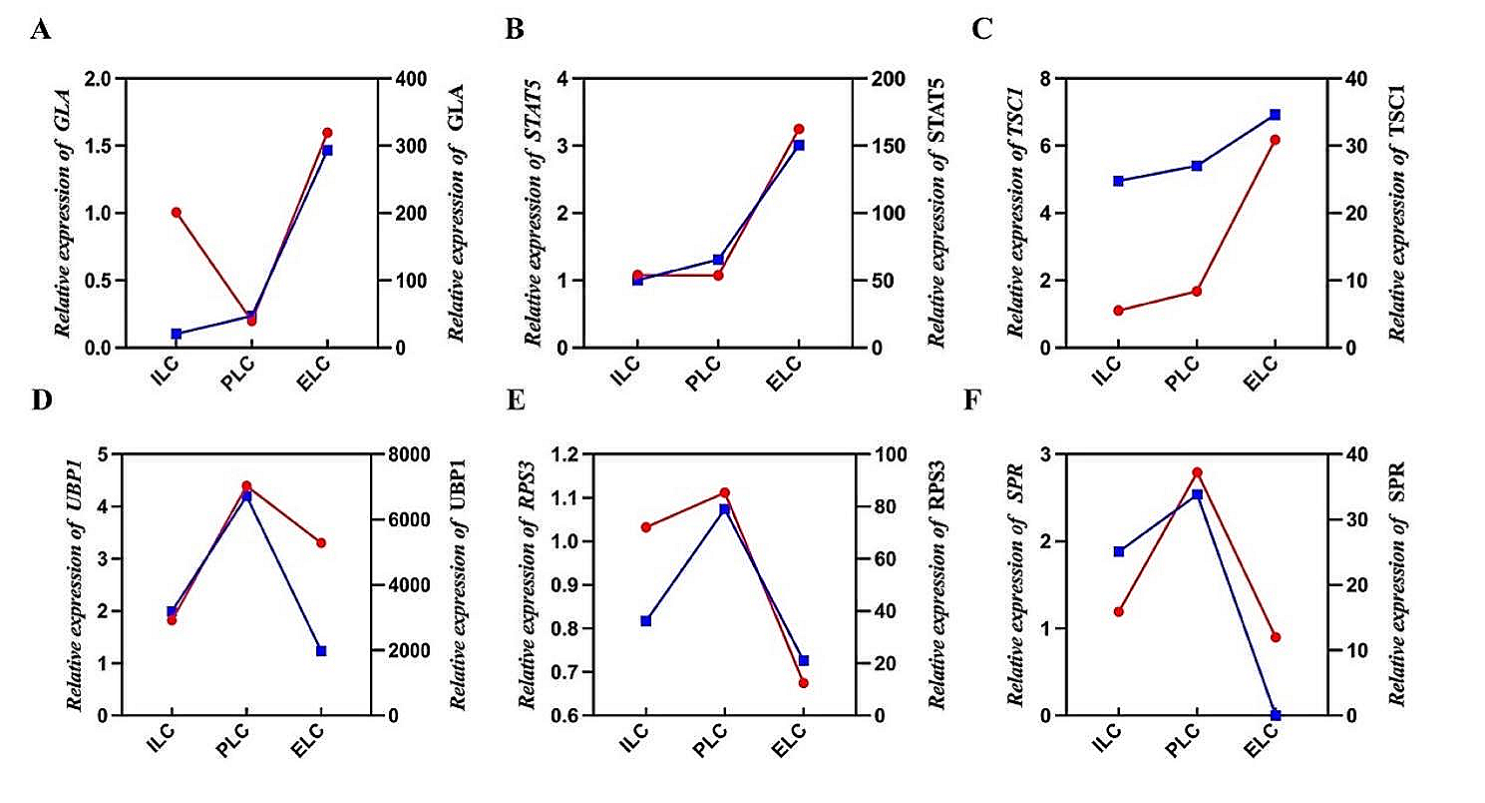
qRT-PCR verification using 6 selected DEPs. (18 S rRNA was selected as the internal reference gene. The results are shown as the means ± SEM of three replicates. The red line is the mRNA expression trend, and the blue line is the protein expression trend determined by sequencing)

To confirm the validity and reliability of sequencing, RT-qPCR was used to determine the transcript abundance of 6 selected DEPs at the three stages of laying cycle. As shown in Fig. 7, the qPCR results showed that the mRNA expression trend of each gene was highly correlated with the sequencing results.

## Discussion

Goose is an important economic livestock product in the Chinese agricultural industry, as it provides eggs, meat and feathers. The reproductive performance of the male breeding has an essential economic effect on the poultry industry and occupies a significant position in the entire poultry breeding system. The main function of male poultry is to produce high-quality sperm to achieve a high egg fertilization rate, so testicles are important reproductive organs for poultry reproduction [13, 14]. Proteomics is a powerful tool for the global analysis of functional networks in biological systems. In contrast to the traditional methods, the emerging data-independent acquisition (DIA) strategy has provided a new dimension for unlabeled quantitative proteomics ^[15]^. In this study, we performed a comprehensive evaluation of the proteomic profile in the testicles of the Jilin white goose ganders between three periods of the laying cycle (PLC vs. ILC, ELC vs. ILC and ELC vs. PLC). A total of 1753 differently expressed proteins of the Jilin white goose testicles were identified by LC-MS/MS based on proteomic strategy. Further, KEGG enrichment analysis, GO annotation and COG analysis were carried out to elucidate the biological implications of the discovered DEPs. Go and COG annotations from Jilin white goose testicle proteomes allow us to predict the potential functions of the identified DEPs. Most of the DEPs were enriched in the GO and COG terms, which were found to be related to the cellular process, metabolic process, regulation of microtubule cytoskeleton organization, structural molecule activity, calcium-dependent phospholipid binding, proteasome complex, calcium ion binding, translation, ribosomal structure and biogenesis and signal transduction mechanisms. The findings suggested that most of the DEPs were involved in the calcium signaling pathway and metabolic pathway, which indicates the presence of active sperm and the occurrence of acrosome reactions in the testis at different reproductive stages [16]. Also, it has been widely reported that germ cells utilize various metabolic pathways for energy production in their different developmental stages [17, 18] and energy metabolism is a key process for the maintenance of sperm development [19]. Further, we demonstrated by analyzing the KEGG pathway that several of the differentially expressed proteins were enriched in the JAK-STAT, MAPK, Notch, mTOR and GnRH signaling pathways and, in the lysosome, steroid hormone biosynthesis, steroid biosynthesis and metabolic pathways in the three comparison groups. These pathways are strongly related to testicular development and the cyclic changes in the testes during the reproductive cycle. The JAK-STAT pathway has been deeply reported to be involved and regulated the major events of male reproduction, such as germline sexual development, germline stem cell maintenance and sperm functions like capacitation and motility [20]. In testis, the majority of male reproductive processes, including sperm maturation and sexual development, are mediated by the JAK-STAT pathway [21]. Additionally, it has been shown that the JAK-STAT pathway is involved in the processes of gametogenesis and the creation and renewal of spermatogonia stem cells, which are crucial for maintaining male fertility [22]. We noticed that mTOR and STAT5B were the most upregulated DEPs in the JAK-STAT pathway. The STAT5B is a member of the signal transducer and activator of the transcription factors family (STAT) [23] and is known to be expressed in the Leydig cell [24], which are responsible for testosterone secretion, a crucial hormone in male reproduction and sperm production. It has been widely known that the regulation of steroidogenesis includes multiple cytokines, growth factors and hormones, such as growth hormone (GH), which is essential for male reproductive function [25]. Mercier et al. [26] found that the treatment of Leydig cell lines in male mice with GH could activate the STAT5B transcription factor by activating the JAK kinases, which then stimulate the Star promoter alone and in cooperation with the transcription factor cJUN, leading to increased steroid hormone production. Interestingly, in our proteomic data, we found that this STAT5B protein is upregulated in the JAK-STAT pathway in the PLC vs. ILC group and in contrast, STAT5B was downregulated in the ELC vs. PLC group (supplementary data), which suggests that STAT5B may have a great potential in goose male reproduction by influencing Leydig cell steroidogenesis, stimulating testosterone secretion and sperm production during the critical stages of laying cycle. For ganders reproduction, the peak of the laying cycle (PLC) stage is characterized by the incensement of testosterone concentrations, which remain higher in the breeding season to nourish the development of germ cells, including spermatogonia, spermatocytes and spermatids in the seminiferous tubules, which led to the incensement of sperm numbers especially during this reproductive stage [27]. It has been reported that testosterone concentration reached peak levels in the onset of the breeding season and subsequently declined in Magang ganders [28]. As the STAT5B protein was upregulated in the JAK-STAT pathway in the comparison group PLC vs. ILC, it is speculated that this pathway, including the STAT5B protein may be related to the secretion of testosterone during this stage and the augmentation of sperm number necessary for egg fertilization during the peak of the laying cycle. Also, it is reported that upregulating Jak2/Stat5b signaling leads to enhanced transcription of the genes required for testosterone synthesis [29]. However, mTOR is associated with spermatogenesis by regulating spermatogonia proliferation and involved in the maintenance and restructuring of the blood-testis barrier [30], a crucial event in the seminiferous epithelium cycle [31, 32]. Several reports have demonstrated that mTOR regulates glucose consumption and redox balance in Sertoli cells and is related to male fertility [33]. In our data, this protein was also upregulated in the mTOR signaling pathway in the PLC vs. ILC group. The mTOR signaling is intimately linked with male reproduction regulation and is involved in many stages of male reproduction and several studies on the mTOR signaling in the testis have focused on the spermatogonial stem cells and Sertoli cells, in which it play an important role in the maintenance of these cells, as well as in regulating the redox balance and metabolic activity of Sertoli cells, which play an essential role in nutritional support during spermatogenesis and controlling male fertility [34, 35]. During the first period of the reproductive cycle, the production of sperm is essential for male reproductive traits. The mTOR protein and especially, mTOR signaling are widely known to be involved in sperm production, and in our data, the upregulation of this protein during the initial stages of the reproductive cycle may be strongly related to the increase in sperm numbers and the changes in testicles during the reproductive cycle. Both the mTOR and RRAGA/B proteins were upregulated in this pathway in the PLC vs. ILC group, which may indicate that this pathway could be involved in goose male reproduction in the early phase of the reproduction cycle. It has also been reported that the RRAGA/B proteins interact with the mTORC1 complex, are known as proteins related to male fertility, especialy in spermatozoa and are found to exist in the head and tail of spermatozoa [36]. Remarkably, we noticed that these proteins were downregulated in the mTOR signaling in the ELC vs. PLC group, suggesting their involvement in goose male fertility and spermatozoa production at the critical stage of the laying cycle. Our findings also demonstrated that several other interesting pathways were enriched in testicles tissues of the Jilin white goose, including the MAPK [37], Notch [38], GnRH signaling pathways [39], and other pathways related to reproductive hormone secretion and metabolic, which suggests that goose male reproduction is regulated with multiple signaling pathways and proteins that were widely studied to be associated with reproduction. It is worth noting that the metabolic pathways in the KEGG enrichment were significantly enriched, which suggested that may are cellular differences in testicular tissue in Jilin white geese with high and low sperm motility, and many of the DEPs identified may play a function in controlling cell metabolism, thereby involving in the regulation of sperm motility in Jilin white geese.

In summary, we investigated the proteome profiles of testicle tissues from Jilin white goose during three different stages of the laying cycle. Some proteins were enriched in different pathways related to reproduction, including MAPK, Notch, GnRH, JAK-STAT, mTOR signaling pathways and pathways associated with reproductive hormone secretion and metabolic. However, the functional significance of these differently expressed proteins needs further investigation. Our findings might provide comprehensive protein expression information that can facilitate the understanding of reproductive biology and improve the reproductive performance of Jilin white goose ganders.

## Conclusions

In conclusion, in this study, we used the DIA strategy to sequence the differential proteome related to the testicular development of Jilin white goose at three stages of the laying period (ILC, PLC and ELC) and conducted a detailed analysis of the dynamic changes in the proteome during this process. Results showed that the DEPs were mainly enriched in several pathways related to testicular development, as in the PLC vs. ILC group, the protein functions are mainly concentrated in Renin secretion, Lysosomes, SNARE interactions in vesicular transport, the p53 signaling pathway and in the ELC vs. ILC group, the protein functions are mainly concentrated in pathways related to metabolism. To summarize, our findings will provide a new insight into the complex molecular mechanism underlying the developmental characteristic of testicles in Jilin white goose.

### Methods

#### Experimental animals

The Jilin white goose population raised in the provenance base of Jilin Agricultural University was selected.

### Sample collection

Three Jilin white geese males in ILC (215 days), PLC (265 days) and ELC (315 days) were selected and sacrificed by cervical dislocation. Both the left and right testicles of each goose were collected for cryopreservation and samples were used for subsequent proteome sequencing and RT-qPCR verification.

### HE staining

Sections were immersed in xylene for dewaxing twice for 5 min each time. Then soak in 100%, 90%, 70% ethanol and distilled water for 2 min each. Dyeing was carried out according to the instructions of the HE staining kit, and finally, the slides were sealed with neutral resin glue and observed under a microscope after drying. The number germ cells were counted using the ImageJ software and Prism 10 for data visualization.

### Protein sample preparation and high PH reverse phase separation

1) Samples were transferred to lysis buffer (2% SDS, 7Murea, 1 mg/mL protease inhibitor cocktail) and homogenized in ice using an ultrasonic homogenizer for 3 min. The homogenate was centrifuged at 15,000 rpm for 15 min at 4 °C and the supernatant was collected.

2) Use the Pierce™ BCA Protein Assay Kit (catalog#23,227 Thermo Fisher Scientific, MA, USA) to measure the protein concentration of the supernatant according to the procedure shown in the instruction manual.

3) Dilute 50 µg protein to 50 µL, add 1 µL of 1 M DTT, and incubate at 55 °C for 1 h. Then add 5 µL of 1 M iodoacetamide (IAA) and keep it in the dark at room temperature for 1 h. Finally, 300 µL of pre-cooled acetone was added to precipitate for 2 h and digested with trypsin (catelog#VA9000, Promega) overnight.

4) All sample peptide mixtures were re-dissolved in buffer A (buffer A: 20 mM ammonium formate aqueous solution, adjusted to pH 10.0 with ammonia) to connect the reverse column (XBridge C18 column, 4.6 mm) with the Ultimate 3000 system (catelog#IQLAAAGADGFAMIMZZZ, Thermo Fisher Scientific, MA, USA) × 250 mm, 5 μm, for high pH separation, linear gradient used for separation, 5% B to 45% B within 40 min (B: 80% ACN with 20 mM ammonium, ammonia adjusted to pH 10.0). The column was equilibrated under initial conditions for 15 min. Six fractions were collected, and each fraction was dried in a vacuum concentrator.

### Nano-LC-MS/MS analysis and proteomics data processing

Peptides separated by liquid chromatography were analyzed by mass spectrometry and data were collected by the DDA method. The collected results were used to build the database. Formal testing used DIA to collect the data, with one sample used for each sample, and the collected data was quantitatively analyzed by DDA combined with the database.

Desalted and lyophilized peptides were redissolved in 0.1% formic acid solution and equipped with online LC-MS/MS for analysis. Mass spectrometer parameter settings were as follow: a complete MS scan (350–1500 m/z) was acquired in the positive ion mode at a resolution of 120,000, an automatic gain control (AGC) target value of 4 × 10^5^, a maximum ion accumulation time of 50 ms and dynamic exclusion time of 30 s. The HCD-MS/MS was as follow: full scan at a resolution of 15,000, AGC target was 5e4 and a maximum ion accumulation time of 35 ms. After, 30 µL of buffer A (A: 0.1% formic acid aqueous solution) was added to each sample to make a suspension and 9 µL of each was extracted. Following, a 1 µL of 10× iRT peptide was added, mixed and separated in nanoLC, and then an online electrospray TA analysis was conducted. The acetonitrile gradient elution procedure was the same as for DDA mass spectrometry to ensure the same protein retention times for DDA library construction and DIA mass spectrometry analysis.

Mass spectrometer parameters were as follow: a complete MS scan with a range of 350–1500 m/z at a resolution of 120,000, an automatic gain control (AGC) target value of 4 × 10^6^ and a maximum ion accumulation time of 50 ms. The HCD-MS/MS were collected as follow: full scan at a resolution of 30,000, AGC target was 1e6, the normalized collision energy was 32 eV and energy gain at 5%. For variable window detection 60 windows were set, overlapping serial ports were placed and each window overlaps were of 1 m/z.

### GO and KEGG pathway enrichment analysis of DEPs

Gene Ontology (GO) enrichment analysis is an internationally standardized protein function classification system (available online: http://www.geneontology.org/), was used to perform functional annotation of differentially expressed proteins. Additionally, Kyoto Encyclopedia of Genes and Genomes (KEGG), which is the main public pathway-related database (available online: http://www.geneontology.org.) was used to further identifier the significantly enriched pathways in DEPs.

### Quantitative real-time PCR (RT-qPCR) validation of sequencing data

The quantitative real-time PCR experiments were performed in an ABI 7500 PCR.

machine (Thermo Fisher, USA) using TransStart® Green qPCR SuperMix (catelog#AQ101-02, TransGene). Each treatment group contained three biological replicates and each sample was tested for relative expression three times. 18 S rRNA was selected as the internal reference gene, and the gene expression level was calculated by the 2^−ΔΔCT^ method [40]. All primers are listed in Table 2.

**Table 2:** Quantitative Real‑Time PCR primer information

Gene	Species	Primer Sequence	Tm
*GLA*	*Anser cygnoides domesticus*	GAAGGCCCATCGTGTACTCC AGCCCGAAGTTCCCAATCAC	60.18 60.32
*STAT5*	*Anser cygnoides domesticus*	CCAAGTACTACACGCCGGTT GATGCGCTGACAAACTCTGG	60.04 59.55
*TSC1*	*Anser cygnoides domesticus*	CCATGAACTGGACCCACGAA ACAGGGTTTCCGAGATGCAG	59.96 60.04
*UBP1*	*Anser cygnoides domesticus*	ATCACCCTCTGAACTGGCTC CAATTGGCCACAGTGACTCG	59.09 59.48
*RPS3*	*Anser cygnoides domesticus*	GCAGGGTGTGTTGGGAATCA GGGTTCCACGATGCTGACAT	60.54 60.39
*SPR*	*Anser cygnoides domesticus*	GCTCATAGACTGCTCCGTGT TCAGACGTTGTAGAAGTCCACG	59.54 60.03
*18 S rRNA*	*Anser cygnoides domesticus*	GCATGGCCGTTCTTAGTTGG GAACGCCACTTGTCCCTCTA	59.55 59.39

### Protein-protein interaction (PPI) network analysis

To predict and identify putative protein-protein interaction networks associated with testicular development, Cytoscape Plug-in Network Analyzer was performed, and the BiNGO plug-in was used as a Cytoscape plug-in for retrieving GO terms. The PPI network was generated from DEGs and differentially expressed proteins related to testicular development. In the network, protein represents the node and edges represent the interaction between nodes. However, the PPI interactions with a composite score (0: lowest confidence; 1: highest confidence) higher than 0.7 were used for further network analysis.

### Electronic supplementary material

Below is the link to the electronic supplementary material.

Supplementary Material 1

## Data Availability

The mass spectrometry proteomics data have been deposited to the Proteome Xchange Consortium (http://proteomecentral.proteomexchange.org) via the iProX partner repository [41, 42] with the dataset identifier PXD046487.
