# Supplementary material for: Protein profile analysis of Jilin white goose testicles at different stages of the laying cycle by DIA strategy
Source: BMC Genomics. 2024 Apr 1;25:326. doi: 10.1186/s12864-024-10166-9 (PMC10986116; doi:10.1186/s12864-024-10166-9)
Supplement: Supplementary file 1 — Supplementary Material 1 [file 12864_2024_10166_MOESM1_ESM.docx]

Table S1. Top 10 DEPs of PLC vs. ILC

| **Upregulated** | **Downregulated** |
| --- | --- |
| Desmin  Ribonuclease P/MRP subunit p38  Lamin A/C  Ig-like domain-containing protein  Ubiquinol-cytochrome-c reductase complex assembly factor 2  von Willebrand factor A domain containing 1  KIAA1045 RING finger domain-containing protein  Uncharacterized protein  beta-N-acetyl hexosaminidase  GDP-D-glucose phosphorylase 1 | Inositol 1,4,5-trisphosphate receptor  Coiled-coil domain containing 28A  AT-rich interactive domain-containing protein 3  Zinc finger protein 706  C2H2-type domain-containing protein  Cms1 ribosomal small subunit homolog  GATOR complex protein NPRL3  ERCC excision repair 6 like, spindle assembly checkpoint helicase  SRY-box transcription factor 9  Adiponectin receptor 1 |

Table S2. Top 10 DEPs of ELC vs. ILC

| **Upregulated** | **Downregulated** |
| --- | --- |
| WH2 domain-containing protein  BTB domain containing 7  GAL3A protein  Collagen type XXVIII alpha 1 chain  FRY like transcription coactivator  CTBP2 protein  [histone H3]-trimethyl-L-lysine (27) demethylase  Nuclear receptor coactivator 4  Myosin light chain 1  Glycylpeptide N-tetradecanoyl transferase | Spermatogenesis associated 20  Carboxymuconolactone decarboxylase-like domain-containing protein  Albumin  Cullin 4A  Proteasome assembly chaperone 2  Histone acetyltransferase type B catalytic subunit  Small nuclear ribonucleoprotein Sm D3  Protein MEMO1  40S ribosomal protein S15a  Small nuclear ribonucleoprotein E |

Table S3. Top 10 DEPs of ELC vs. PLC

| **Upregulated** | **Downregulated** |
| --- | --- |
| Testis specific serine kinase 6  Serpin domain-containing protein  TATA-box binding protein associated factor 5  Cleavage and polyadenylation specificity factor subunit 4  Fibronectin type-III domain-containing protein  Cytidine deaminase  ASXL transcriptional regulator 2  XK-related protein  Anaphase-promoting complex subunit 5  [histone H3]-trimethyl-L-lysine (27) ademethylase | Desmin  Hemoglobin subunit epsilon 1  Ornithine aminotransferase  Sperm acrosome associated 1  PurE domain-containing protein  Tumor protein p53 inducible protein 3  Ig-like domain-containing protein  Hydroxypyruvate isomerase (putative)  Cholecystokinin  Protein phosphatase 1 regulatory subunit 12B |


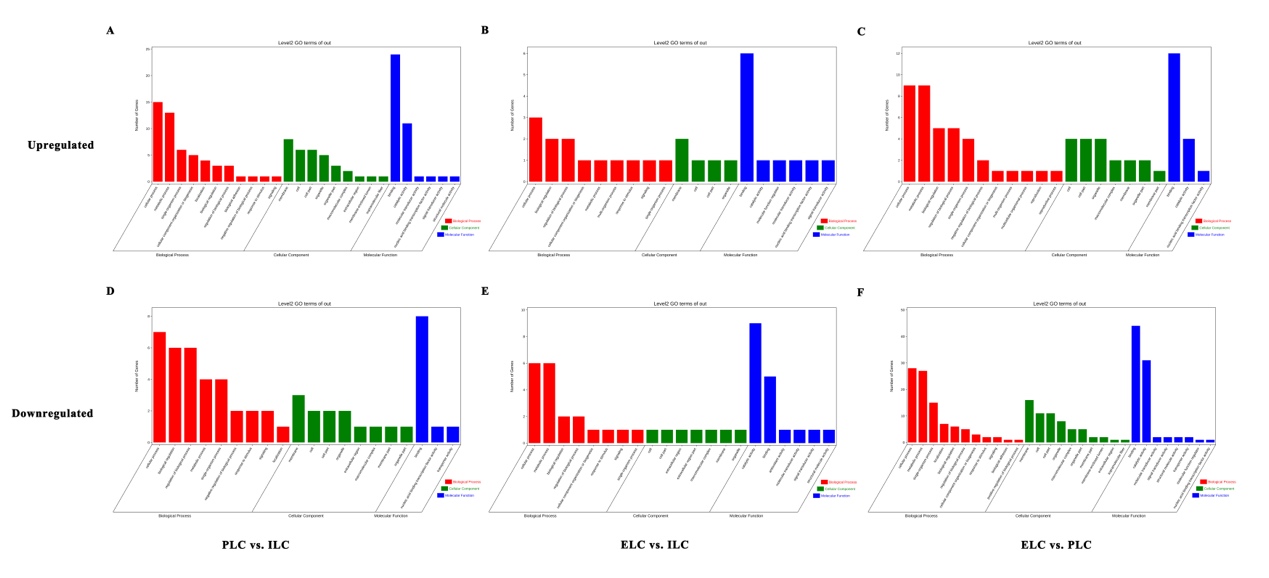


**Fig S1. GO enrichment analysis of differentially expressed proteins (DEPs) in Jilin white goose testicles at different stages of the laying cycle.** (A-C) shows the comparison group of ILC, PLC and ELC. The results are elaborated into three major groups: Biological process, cellular component and molecular function. The Y-axis shows the percentage of proteins, while the X-axis displays the second level term of the gene ontology.

**
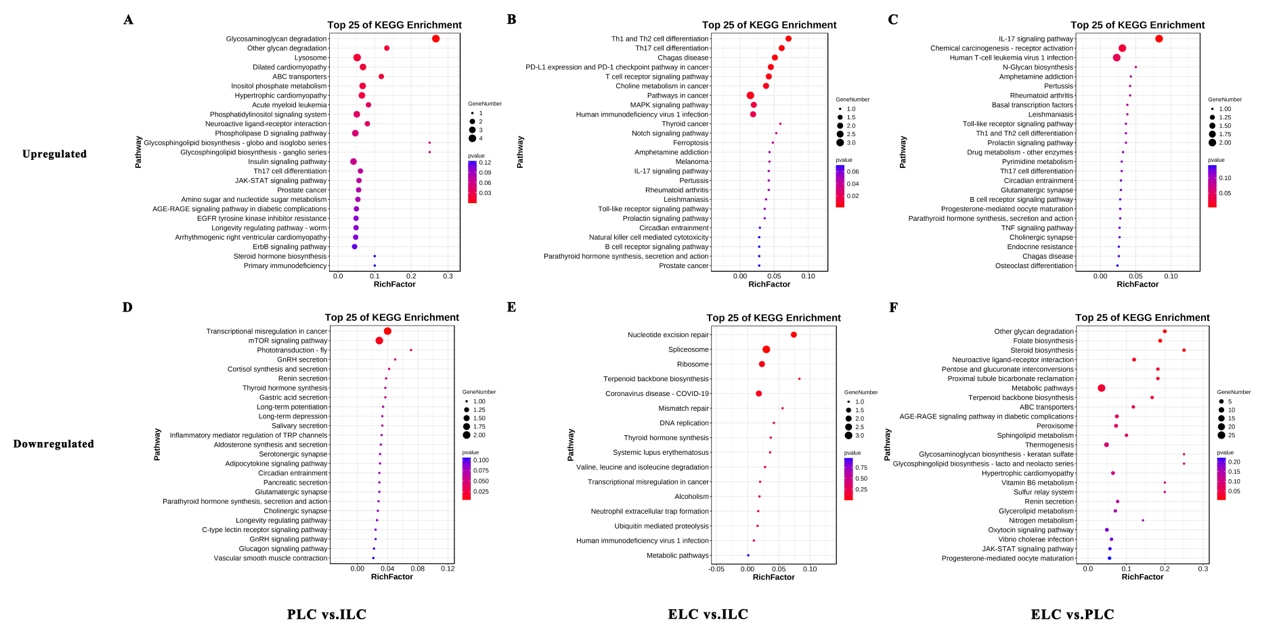
**

**Fig S2. KEGG pathway enrichment analysis of differentially expressed proteins (DEPs) in Jilin white goose testicles at different stages of the laying cycle.** (A-C shows the comparison group of ILC, PLC and ELC. The abscissa represents the number of enriched proteins, and the ordinates represent signal pathways**.**)
